# Cognitive behavioural therapy versus multidisciplinary rehabilitation treatment for patients with chronic fatigue syndrome: study protocol for a randomised controlled trial (FatiGo)

**DOI:** 10.1186/1745-6215-13-71

**Published:** 2012-05-30

**Authors:** Desirée CWM Vos-Vromans, Rob JEM Smeets, Leonie JM Rijnders, René RM Gorrissen, Menno Pont, Albère JA Köke, Minou WMGC Hitters, Silvia MAA Evers, André J Knottnerus

**Affiliations:** 1 Revant Rehabilitation Centre Breda, Brabantlaan 1, 4817, JW, Breda, The Netherlands; 2 Department of Rehabilitation Medicine, Research School CAPHRI Maastricht University, P.O. Box 616, 6200, MD, Maastricht, The Netherlands; 3 Adelante Centre of Expertise in Rehabilitation and Audiology, P.O. Box 88, 6430, AB, Hoensbroek, The Netherlands; 4 Reade Centre for Rheumatology and Rehabilitation, P.O Box 58271, 1040, HG, Amsterdam, The Netherlands; 5 Rehabilitation Centre Blixembosch, P.O. Box 1355, 5602, BJ, Eindhoven, The Netherlands; 6 Department of Health Organization Policy and Economics, Research School CAPHRI Maastricht University, P.O. Box 616, 6200, MD, Maastricht, The Netherlands; 7 Department of General Practice, Research School CAPHRI Maastricht University, P.O. Box 616, 6200, MD, Maastricht, The Netherlands

**Keywords:** Chronic fatigue syndrome, CBT, Multidisciplinary rehabilitation treatment, Cost-effectiveness, Fatigue, Quality of life

## Abstract

**Background:**

Patients with chronic fatigue syndrome experience extreme fatigue, which often leads to substantial limitations of occupational, educational, social and personal activities. Currently, there is no consensus regarding the treatment. Patients try many different therapies to overcome their fatigue. Although there is no consensus, cognitive behavioural therapy is seen as one of the most effective treatments. Little is known about multidisciplinary rehabilitation treatment, a combination of cognitive behavioural therapy with principles of mindfulness, gradual increase of activities, body awareness therapy and pacing. The difference in effectiveness and cost-effectiveness between multidisciplinary rehabilitation treatment and cognitive behavioural therapy is as yet unknown. The FatiGo (Fatigue-Go) trial aims to compare the effects of both treatment approaches in outpatient rehabilitation on fatigue severity and quality of life in patients with chronic fatigue syndrome.

**Methods:**

One hundred twenty patients who meet the criteria of chronic fatigue syndrome, fulfil the inclusion criteria and sign the informed consent form will be recruited. Both treatments take 6 months to complete. The outcome will be assessed at 6 and 12 months after the start of treatment. Two weeks after the start of treatment, expectancy and credibility will be measured, and patients will be asked to write down their personal goals and score their current performance on these goals on a visual analogue scale. At 6 and 14 weeks after the start of treatment, the primary outcome and three potential mediators—self-efficacy, causal attributions and present-centred attention-awareness—will be measured. Primary outcomes are fatigue severity and quality of life. Secondary outcomes are physical activity, psychological symptoms, self-efficacy, causal attributions, impact of disease on emotional and physical functioning, present-centred attention-awareness, life satisfaction, patient personal goals, self-rated improvement and economic costs. The primary analysis will be based on intention to treat, and longitudinal analysis of covariance will be used to compare treatments.

**Discussion:**

The results of the trial will provide information on the effects of cognitive behavioural therapy and multidisciplinary rehabilitation treatment at 6 and 12 months follow-up, mediators of the outcome, cost-effectiveness, cost-utility, and the influence of treatment expectancy and credibility on the effectiveness of both treatments in patients with chronic fatigue syndrome.

**Trial registration:**

Current Controlled Trials ISRCTN77567702.

## Background

In chronic fatigue syndrome (CFS), patients experience extreme fatigue that is medically unexplained. Many patients feel limited in their daily activities, and are not able to work at all or as much as they did before their CFS started [1]. Social and leisure activities are reduced in most patients, and quality of life is low [2].

Of the current definitions of CFS [3], we use the definition of the US Centers for Disease Control and Prevention (CDC-94): a persistent or relapsing unexplained fatigue, of new or definite onset and lasting for at least 6 months, in which fatigue is not the result of an organic disease or ongoing exertion. Rest does not alleviate the fatigue, and there is substantial limitation of occupational, educational, social and personal activities. To support the diagnosis, four or more of the following symptoms should be present for more than 6 months: impaired memory or concentration, sore throat, tender cervical or axillary lymph nodes, muscle pain, pain in several joints, new headaches, unrefreshing sleep or malaise after exertion [4]. Three studies from the UK and the USA, using this definition, show prevalence rates between 0.23 and 0.50% [5-7]. In The Netherlands approximately 30,000-40,000 patients suffer from CFS [8].

The pathophysiology of CFS is unclear. Researchers have considered somatic (e.g. viral infection, dysfunction of the central nervous system, immune dysfunction and neuroendocrine responses) and psychosocial hypotheses. A commonly used hypothesis relates CFS to stress. According to Van Houdenhove [9], patients have a reduced effort tolerance, which might be interpreted as a fundamental failure of the stress system after a period of severe or prolonged physical and/or psychosocial stress in vulnerable individuals. The failure of the stress system may lead to disturbances in the nervous, hormone and immune systems. Many studies have tried to investigate different parts of these systems, but the precise mechanisms are still unclear [3,10]. Although there is no consensus on the pathophysiology of CFS, most researchers and clinicians believe that the aetiology is multifactorial [3,11].

Different predisposing, precipitating and perpetuating factors play an important role in the aetiology of CFS [3]. Lifestyle and personality characteristics like neuroticism and introversion are examples of predisposing factors for developing CFS [3,12]. Acute physical of psychological stress are precipitating factors that may trigger the onset of CFS [13]. Cognitions, beliefs and attributions about complaints and behavioural factors such as persistent avoidance of activities are associated with an increase of symptoms [14]. Other perpetuating factors are a strong belief in a physical cause of the illness, a strong focus on physical sensations and poor sense of control over the complaints. Social processes, for example lack of social support, also contribute to the perpetuation of CFS [15].

Although many patients suffer from CFS, many parts of this syndrome are still unclear and need further research in order to understand the pathophysiology and aetiology, and to improve and customise treatment to individuals in accordance with the different pathophysiology and/or aetiology.

### Relevance

Many studies have investigated the effects of different treatments that are targeted towards one or two aspects of the complaints. Little is known about treatments that are targeted towards more aspects of the complaints and combine different interventions in multidisciplinary settings. Although a few treatments that are targeted towards one or two aspects of the complaints have significant effect on fatigue severity and quality of life, no consensus exists on the treatment of patients with CFS. Many patients try different therapies to overcome their fatigue, varying from pharmacological treatment (for example immunoglobulin therapy and fludrocortisone therapy) to non-pharmacological treatments (for example massage therapy and osteopathy). Several reviews [3,10,16-18] compare different treatments. Immediately post-treatment, cognitive behavioural therapy (CBT) and graded exercise therapy (GET) are the only interventions found to be beneficial in reducing the severity of fatigue symptoms when compared with usual care [18,19]. At medium term (with a maximal of 14 months after baseline), CBT is also more effective than usual care in reducing fatigue severity [18]. The review by Edmonds et al. [19] showed two studies that found no significant difference between GET and treatment as usual/relaxation in the severity of fatigue at medium term. On quality of life, one study investigating the benefits of CBT compared to usual care found no significant difference post treatment [18]. Three studies [20-22] analysed the change in quality of life between GET and treatment as usual, and found that the physical function subscale improved significantly with exercise therapy immediately post treatment. Besides studies in which CBT and GET are compared to usual care, three studies [23-25], compared CBT with other psychological therapies. They provide evidence that CBT was more effective in reducing the severity of fatigue symptoms in CFS patients post treatment, but the evidence of medium- and long-term follow-up was inconsistent [18]. In three other studies [26-28] CBT was compared to GET and showed a lack of difference between both treatments in reducing fatigue levels at post-treatment and at medium-term follow-up [18]. In the randomised trial of White et al. [28], CBT and GET were compared with adaptive pacing therapy (APT) and specialist medical care (SMC) alone. Participants had less fatigue and better physical function after CBT and GET than they did after APT or SMC alone.

Although several studies have shown positive effects on reducing fatigue severity after treatment, some CFS patient groups are negative about CBT, as well as GET [29,30].

At this time, most treatments are targeted towards one or two aspects of the complaints, but various experts [8,18,31] recommend using CBT in combination with other interventions or in a multidisciplinary setting in order to increase treatment effectiveness. To date, only a few studies have reported results of a multidisciplinary approach in peer-reviewed scientific journals. Two uncontrolled studies among young people [32,33] reported positive effects of multidisciplinary interventions. Viner et al. (2004) [32] assessed the outcome of multidisciplinary rehabilitation group treatment (graded activity/exercise programme, family sessions and supportive care) compared with supportive care alone. Results showed positive effects of multidisciplinary rehabilitation treatment on wellness, school attendance and severity of fatigue. A study by Voet et al. (2007) [33] in adolescents with chronic pain and fatigue also showed strong positive effects on fatigue severity, school/work attendance and general health after multidisciplinary rehabilitation treatment. In an uncontrolled study, Torenbeek et al. (2006) [34] evaluated a multidisciplinary group program with a combination of clinical and outpatient treatment in a rehabilitation centre. Patients were coached by a rehabilitation physician, psychologist, physical therapist, social worker, occupational therapist, exercise and sports coach, and a group leader. Positive effects were found on fatigue severity, experienced impairments and physical functioning, post treatment. In another uncontrolled pilot study of Vos-Vromans (2005) in Revant Rehabilitation Centre Breda, in which the MRT was evaluated among 36 patients, the results were promising: fatigue severity and the impact of disease on emotional and physical functioning decreased significantly post treatment and persisted 12 months after start of treatment. Although the results of these studies are promising, conclusions should be drawn carefully, because all studies were uncontrolled. None of the above studies included information about the cost-effectiveness of multidisciplinary rehabilitation treatment, which might facilitate the decision process regarding treatment selection for practitioners as well as policy makers. Therefore, these findings need to be confirmed in randomised controlled trials including an economic evaluation.

In summary, CBT is the most effective treatment at this time; therefore, CBT needs to be compared with a multidisciplinary rehabilitation approach to investigate which treatment is the most effective and most cost-effective. Because effects of CBT on quality of life and other secondary outcomes in the medium and long term are inconclusive, more research is needed to examine how to sustain the treatment effect.

### Aims of the study

The FatiGo trial is designed to address the following primary objectives:

 1. To assess the differences in treatment effect (change between baseline and 6-month follow-up in fatigue severity and quality of life) in patients with CFS between individual multidisciplinary rehabilitation treatment (MRT) and individual cognitive behavioural therapy (CBT).

 2. To assess the differences in long-term treatment effect (change between baseline and 12-month follow-up in fatigue severity and quality of life) in patients with CFS between the two treatments.

 3. To assess the difference in cost-effectiveness and cost-utility between MRT and CBT from both a health care and societal perspective at 12-month follow-up.

 4. To assess the differences in treatment effect in psychological symptoms, self-efficacy, causal attributions, present-centred attention-awareness, impact of disease on physical and emotional functioning, self-rated improvement and life satisfaction between MRT and CBT (at 6- and 12-month follow-up).

The secondary objectives of this trial are:

 1. To assess the influence of patients treatment expectancy and credibility on the effectiveness of treatment.

 2. To assess what baseline factors (other than the assigned treatment) predict a change in fatigue and increase in quality of life in all participants.

 3. To evaluate whether changes in self-efficacy, changes in causal attributions and/ or changes in present-centred attention-awareness (at baseline, 6 and 14 weeks after start of treatment) mediate changes in fatigue severity and changes in quality of life after treatment.

### Hypotheses on the primary objectives

 (1) MRT is more effective than CBT in reducing fatigue severity and increasing quality of life 6 months after start of treatment.

 (2) MRT is more effective than CBT in reducing fatigue severity and increasing quality of life 12 months after start of treatment.

 (3) MRT is more cost-effective than CBT when data on medical and non-medical costs are compared over a 12-month period and shows a higher cost-utility.

 (4) MRT is more effective than CBT in increasing self-efficacy, non-physical attributions, present-centred attention-awareness and life satisfaction, and is more effective in decreasing the impact of disease on physical and emotional functioning and decreasing the psychological symptoms. Self-rated improvement is significantly higher in MRT than in CBT (at 6- and 12-month follow-up).

## Methods

### Design

**Figure 1:**
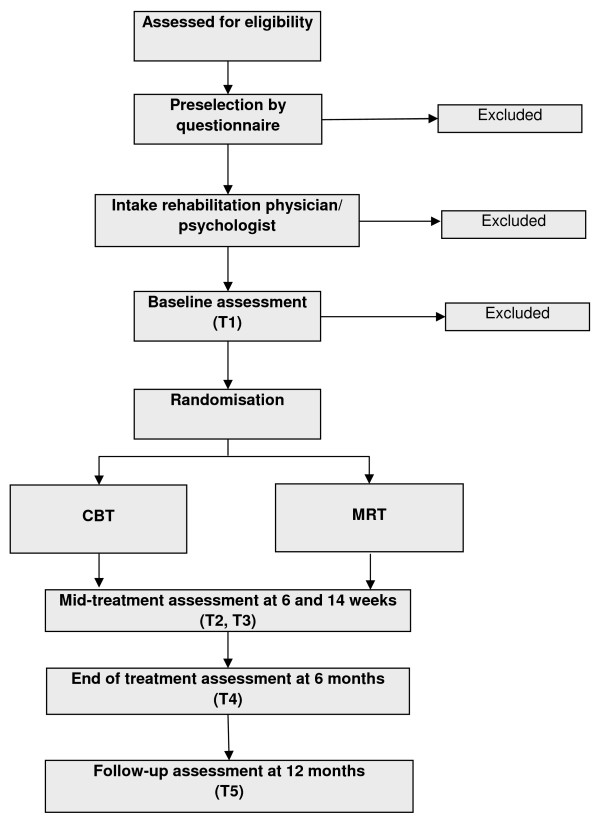
Flowchart of trial design.

A two-arm, pragmatic, randomised, multi-centre controlled trial of patients with CFS, with follow-up of 1 year (see Figure 1). The RCT includes both an effectiveness study as well as an economic evaluation.

### Setting

The study takes place in The Netherlands in four rehabilitation centres: Revant Rehabilitation Centre Breda (RRCB), Rehabilitation Centre Blixembosch in Eindhoven (RCB), Reade Centre for Rheumatology and Rehabilitation in Amsterdam (RCRR) and Adelante Rehabilitation Centre in Hoensbroek (ARC). Patients are referred to the trial by their general practitioner or a medical specialist. Patients are treated individually in an outpatient setting.

### Ethical approval

Ethical approval for the FatiGo trial was provided by the Research Ethics Committee of Rotterdam (reference 2008/22).

### The participants – inclusion and exclusion criteria

Subjects are patients with CFS referred to RRCB, RCRR, RCB and ARC. Patients are included if the following inclusion criteria are met:

 1. The participant has given written informed consent.

 2. The participant meets the CDC- 94 criteria for CFS.

 3. The Checklist Individual Strength (CIS)-fatigue score is 40 or more.

 4. The participant is willing to participate in a treatment that is set up to change behaviour.

 5. The participant is aged between 18 years and 60 years old.

 6. The participant is able to speak, understand and write the Dutch language.

Exclusion criteria are:

 1. Any medical condition that can explain the presence of chronic fatigue.

 2. A psychotic, major or bipolar depressive disorder (but not an uncomplicated depression).

 3. Dementia

 4. Anorexia or bulimia nervosa

 5. Alcohol and/or drug abuse

 6. Severe obesity (BMI ≥ 45)

 7. Pregnancy

 8. Previous or current CBT or MRT with regard to CFS.

 9. More than 1 h travelling time to the nearest participating rehabilitation centre.

### Screening for participation

When a patient is referred to one of the four rehabilitation centres, the research assistant screens the information provided by the referring general practitioner or medical specialist. The research assistant sends the patient information on the study and asks the patient to fill in the Checklist Individual Strength to measure fatigue and the Anxiety and Depression Scale (HADS) [35]. If the patient has a CIS fatigue score of 40 or more, the patient is invited for an intake with the rehabilitation physician. All patients are screened by a rehabilitation physician to check the in- and exclusion criteria. The rehabilitation physician will verify whether an extensive physical examination and laboratory research according the guidelines for chronic fatigue syndrome by the Dutch Diagnostic Compass [36] have been done by a general practitioner, consultant in internal medicine, neurologist or psychiatrist to exclude any underlying illness. If the rehabilitation physician needs a second opinion to decide whether a patient meets the in- or exclusion criteria or when the HADS depression score is 11 or higher, an intake with a psychologist is planned.

The rehabilitation physician explains the procedures of the study, and if someone meets the inclusion criteria and does not meet the exclusion criteria, he asks the patient to sign a (written) agreement. The research assistant contacts the patient after 1 week to make an appointment for signing the informed consent form and for the baseline assessment.

### The interventions

The two interventions to be compared, MRT and CBT, take 6 months to complete.

Three elements are incorporated in both treatment groups:

 1) Modification of dysfunctional beliefs regarding illness symptoms and activity, and development of more adequate and effective coping strategies.

 2) Gradual increase of activities.

 3) Normalisation of sleep/wake rhythm.

These elements are incorporated in both treatments in a different way (see below).

#### Individual cognitive behavioural therapy (CBT)

CBT is a psychotherapeutic approach in which elements of behavioural therapy and cognitive therapy approaches are incorporated. In CBT, a model of perpetuating cognitions and behaviour of CFS [14] is used to explain the persistence of CFS. This model shows that high physical attributions will decrease physical activity and increase fatigue and functional impairment. This model also explains that a low level of sense of control over symptoms and focusing on physical sensations have a direct causal effect on fatigue severity and functional impairment. A perceived lack of social support also increases the fatigue severity and functional impairment. These perpetuating factors (high physical attributions, decreased physical activity, low level of sense of control, focusing on physical sensations and perceived lack of social support) are the focus of the intervention in CBT [37,38].

CBT is divided into three phases:

 1) Intake

 2) Gradual reactivation

 3) Prevention of relapse

 1) Intake

During the intake phase (four sessions in 4 weeks), the cognitive behavioural therapist gets acquainted with the patient. The patient is asked about: the cause and course of the complaints, the present complaints, illness beliefs and illness behaviour, coping, social interactions/participation, and the expectations and personal goals of the patient. The therapist tries to determine the patient’s activity level by asking about activities during the day and week, and categorises the patient into a relatively active patient or a patient with a low activity pattern. The therapist explains the model of perpetuating cognitions and behaviour of CFS, and how to overcome CFS by changing patterns of thinking and changing behaviour.

 2) Gradual reactivation

 Graded exercise therapy (GET) is used to gradually increase physical activity. The patient follows a schedule to gradually increase activities at home (walking and bicycling). The schedule is provided by the therapist in accordance with the patient’s personal goals. The patient has to increase his/her activities at home and receives feedback afterwards during the next therapy session. If needed, schedules are made to increase social and/or mental activities as well. Another important subject during gradual reactivation is the balance between different activities and the patient’s personal responsibility to see to it.

 In the dialogues with the therapist and by doing exercises at home, the patient is taught to change negative beliefs regarding symptoms of fatigue, self-expectations and self-esteem. Specific lifestyle changes are encouraged if deemed appropriate.

 Sleep/wake rhythm: the patient is encouraged to change the sleep/wake rhythm immediately at the start of treatment into a regular sleep/wake rhythm. Sleeping during the day is not allowed.

 In accordance with the principles of GET, a plan to return to work will be made.

 3) Prevention of relapse:

If activities are increased and the sleep/wake rhythm is normalised, the patient is encouraged to unsettle him-/herself and to cope with these disturbances by applying the things he/she learned during therapy. Personal goals are evaluated and relapse prevention is addressed.

The patient assigned to this group will attend 16 individual therapy sessions, spread out over 6 months with a psychologist or behavioural therapist. The first 6 weeks, the patient has weekly contact with the therapist, followed by once every 2 weeks for the next 20 weeks. The CBT protocol is fixed and different for relatively active patients and patients with a low activity pattern [37,38]. In the treatment for the relatively active patient, the patient learns to spread out activities during the day and to vary different activities during the day. The patient learns to be active within physical and mental boundaries to overcome overburdening. With the use of cognitive therapy, cognitions and behaviour that may lead to overburdening (like not accepting boundaries in activity, and having high expectations) are the primary focus of treatment. After reaching the baseline (without peaks in complaints of the CFS) there will be a gradual increase of activities. For patients with a low activity pattern, activities will be increased from the beginning of therapy.

#### Individual multidisciplinary rehabilitation treatment (MRT)

In multidisciplinary treatment, a biopsychosocial model of CFS is used including biological, physical and psychosocial aspects [10,31]. In the biopsychosocial model of CFS various precipitating, predisposing and perpetuating factors are merged, suggesting that multiple pathways may lead to the causation and persistence of CFS [31]. The protocol of the MRT is not fixed, but varies between patients, depending on the relation between treatable components (precipitating, predisposing and perpetuating factors), present complaints and personal needs of a patient. The focus of treatment can be different for each patient depending on these relations. During treatment every therapist fills in treatment checklists for every patient to register which methods are used.

MRT is divided into three phases:

 1) Observation

 2) Treatment

 3) Prevention of relapse

 1) Observation

During a 2-week observation, therapists (psychologist, social worker, physical therapist and occupational therapist) get acquainted with the patient. During observation, they ask the patient about: the cause and course of the complaints, the present complaints, illness beliefs and illness behaviour, coping, the social environment the patient lives in, expectations and personal goals. The psychologist (two 1-h sessions) further elaborates on the psychological history, present psychological wellbeing, use of medical care including medication, stress factors, cognitions, attitudes and mood (state of mind). The social worker (two 1-h sessions) assesses the social context in which the patient lives (relationships, family and role in a family), work situation and communication. The physical therapist (five 30-min sessions) makes an estimation of the physical condition and the patient’s body awareness. The occupational therapist (four 30-min sessions) aims at ergonomics, lifestyle, day/week schedule and the variety of activities during the day/week. During observation, the treatable components are weighted in relation to the present complaints. If a strong relation exists between these components and the present complaints, these components will be addressed during treatment. In a team meeting, therapists and the rehabilitation physician discuss the components and methods that will be used during the treatment phase. The rehabilitation physician will discuss the conclusions of this meeting with the patient and ask for commitment to the proposed therapy. A treatment contract will be signed by the rehabilitation physician and the patient.

 2) Treatment

Two weeks after ending the observation phase, the treatment phase starts. This phase takes 10 weeks to complete. Depending on the patient goals/needs and the relation between treatable components and present complaints, different methods will be more or less used in the treatment phase. The following methods can be incorporated:

 Body awareness therapy [39,40]: aims to establish an increased awareness and consciousness of the body and its relation to psychological wellbeing. The patient learns to discriminate bodily symptoms other than fatigue and pain and learns to react on these healthy bodily symptoms. The patient will be coached by a physical therapist. Bodyscan, grounding, awareness exercises of the influence of thoughts and emotions on the body are some of the exercises that will be practised during treatment. In the end, the patient will be aware of the relation between the body, its physical function, psychological wellbeing and social interaction, and is able to react on stress in an appropriate way.

 Cognitive behavioural therapy: A psychotherapeutic approach in which elements of behavioural and cognitive therapy approaches are incorporated. CBT facilitates the identification of unhelpful, negative emotion-provoking thoughts, dysfunctional emotions, behaviours and cognitive patterns, and challenges them through a goal-oriented, systematic procedure. The patient learns to identify negative beliefs regarding the symptoms of fatigue, self-expectations or self-esteem, and is encouraged to challenge and change them into new, more realistic, more helpful alternatives.

 Gradual reactivation: At the start of treatment, activities are trained time contingent under close supervision of the physical therapist and occupational therapist. The patient follows schedules to gradually increase activities and receives immediate feedback during treatment when needed. The schedules of fitness exercises and swimming are provided by the physical therapist in accordance with the patient’s personal goals. Another schedule is provided by the occupational therapist in accordance with the patient’s personal goals to increase activity and vary activities at home. In the final phase of treatment, schedules are of less importance and the patient is encouraged to increase activities on his/her own without following a schedule (see pacing).

 Pacing: During the second phase of treatment, the patient is taught to pace his/her activities during the day/week. By developing awareness of healthy bodily symptoms the patient will be able to balance his/her activities (psychological as well as physical activities) before extreme fatigue or pain prevails. The schedule of time-contingent increase is no longer followed. The patient will pace his/her activities based on his/her own experiences.

 Principles of mindfulness. Mindfulness is a non-elaborative, non-judgemental, present-centred awareness in which each thought, feeling or sensation that arises is acknowledged and accepted as it is. The patient learns to self-regulate attention that is maintained on immediate experience, thereby allowing for increased recognition of mental events in the present moment. They also learn to observe the thoughts, emotions and sensations that arise, without making judgements about their truth, importance or value, and without trying to escape, avoid or change them. Regular practice of mindfulness skills increases self-awareness and self-acceptance, reduces reactivity to passing thoughts and emotions, and improves the ability to make adaptive choices [41]. In patients who have been chronically ill, mindfulness skills have a positive effect on depression, mood and activity level [42].

 Normalising of the sleep/wake rhythm. The sleep/wake rhythm will be discussed and with a schedule of 4 weeks will be gradually changed to the sleep/wake rhythm the patient desires. Sleeping during the day will be stopped immediately. If there are problems with the quality of sleep, principles of sleep hygiene are prescribed by the psychologist. Relaxation therapy is used to increase the efficiency of the resting moments during the day and to improve the quality of sleep during the night if needed.

 Social reintegration. Under supervision of the occupational therapist and social worker, the patient is coached to reintegrate into society by making a plan to return to his/her work or school, and to increase their social activities.

 3) Prevention of relapse

Six weeks after ending the treatment phase, the patient will visit the social worker. Thirteen weeks after ending the treatment phase, the patient will visit two therapists of his/her choice who were involved in the previous treatment. Both after-care visits are used to stimulate and motivate the patient to practice at home what he/she has learned during the treatment phase.

Although MRT and CBT have three corresponding aims—modification of dysfunctional beliefs, gradual increase of activities and normalisation of sleep/wake rhythm—many differences can be detected between the two treatments. The main differences are viewed in Table 1.

**Table 1:** Differences between CBT and MRT

**CBT**	**MRT**
Treatment focus on perpetuating factors	Treatment focus depending on the relation between the (precipitating, predisposing and perpetuating) factors and the presented complaints.
Afterwards feedback at next therapy session	Immediately feedback during therapy
Pays no attention to physical sensations	Stimulating awareness of healthy bodily symptoms
CBT	CBT incorporated with principles of mindfulness

### Training the therapists to deliver the interventions

Four rehabilitation teams deliver the multidisciplinary rehabilitation treatment. Each team consists of one or two rehabilitation physicians or physician assistants (under supervision of a rehabilitation physician), one or two psychologists/behavioural therapists, two social workers, two physical therapists and two occupational therapists. Six other psychologists/cognitive behavioural therapists deliver the cognitive behavioural therapy in the four participating rehabilitation centres. The psychologists of the CBT group are not involved in the multidisciplinary rehabilitation treatment and meetings with a supervisor will be organised separately for both groups of psychologists.

#### CBT

All psychologists and behavioural therapists are trained in CBT. A 3-day workshop before the start of the study was held, guided by an external CBT expert, who is acquainted with the CBT protocol [38,43] to ensure that execution of CBT is similar and up to standard in each centre. During the trial seven supervision meetings are organised in which audiotaped sessions provided during the trial are used to evaluate the therapy. Therapists are free to contact their supervisor when questions arise.

#### MRT

Before the beginning of the study, the therapists of RRCB, who work with the protocol for at least 5 years, organised separate workshops for each involved discipline and a multidisciplinary team meeting in which the therapists got acquainted with the MRT protocol. During the trial two disciplinary supervision meetings and two multidisciplinary team supervision meetings will be held. Therapists are free to contact their supervisor when questions arise.

### Recruitment of patients

The inclusion of new patients took place from November 2008 until January 2011. Potential referrers of patients to the four rehabilitation centres were informed about the developments of the trial by newsletters four times during the trial. Several articles on Internet sites and in magazines of patient support groups were published to inform patients about the trial and how they could be referred.

### Outcome measures

#### Primary outcome measures

Primary outcome parameter:

 Fatigue severity is assessed by a subscale of the Checklist Individual Strength (CIS) [44,45]. The subscale consists of eight items, each scored on a 7-point Likert scale (range 8–56). Validity and reliability of the scale are good [44,46].

 Quality of life is assessed by the Short-Form 36 (SF-36) [47]. The SF-36 has eight subscales: physical functioning (10 items), role-physical (4 items), bodily pain (2 items), general health (5 items), vitality (4 items), social functioning (2 items), role-emotional (3 items) and mental health (5 items). Every subscale is transformed into ratings on a scale of 0 (limited in all activities) to 100 (able to carry out vigorous activities). The validity and reliability of every subscale are high [48].

#### Secondary outcome measures

Secondary outcome parameters are:

 1. Psychological symptoms will be measured with the Symptom Check List-90 (SCL-90). The SCL-90 is a multidimensional questionnaire designed to screen for a broad range of psychological problems. The questionnaire consists of 90 items. Each item is scored on a 5-point Likert scale (0 is ‘not at all’ and 4 is ‘extremely’) [45,49]. The items are combined in the following primary symptom dimensions: somatisation, obsessive-compulsive, interpersonal sensitivity, depression, anxiety, anger-hostility, phobic anxiety, paranoid ideation and psychoneuroticism (total score of the SCL-90). The validity and discriminating validity are good [50].

 2. Self-efficacy will be measured with the Self-Efficacy Scale-28 (SES 28) to compare sense of control in relation to CFS complaints [25,45]. The scale consists of seven questions. Items are scored on a 4-point Likert scale. The total score ranges from 7 to 28. A higher score means more sense of control.

 3. Causal attributions will be measured with the Causal Attribution List (CAL) [25]. The CAL assesses whether the patient is likely to attribute complaints to physical or non-physical causes. The list consists of ten questions scored on a 4-point Likert scale. Total subscale scores of physical and non-physical attributions range from 5 to 20. A higher score indicates a stronger conviction.

 4. Present-centred attention-awareness, which is foundational to mindfulness, will be measured with the Mindfulness Attention Awareness Scale (MAAS) [51]. The validity and reliability of the Dutch version of the MAAS are good [52]. The MAAS consists of 15 statements scored on a 6-point Likert scale. The mean total score ranges from 1 to 6. A higher score indicates a greater awareness of present experiences.

 5. A patient’s personal treatment goals will be measured with the Patient-Specific Complaints and Goals questionnaire (PSCG) [53]. The patient selects three activities that he/she perceives as important in his/her daily life and wants to improve. The patient rates the performance of the activity on a 100-mm visual analogue scale (VAS). The left side of the VAS is marked as ‘no problems at all’. On the right side the VAS is marked ‘impossible’. The PSCG is a valid and reliable measure with sufficient responsiveness [53].

 6. Sickness Impact Profile-8 will be used to measure the impact of disease on both physical and emotional functioning [54]. The SIP-8 is derived from the SIP. The SIP8 has eight subscales: home management, mobility, alertness behaviour, sleep/rest, ambulation, social interaction, work, and recreation and pastimes. Psychometric research has indicated that the SIP is reliable and valid [55,56].

 7. Physical activity will be measured by a multisensor armband (Sense Wear Pro Armband; BodyMedia, Inc., Pittsburgh, PA). The armband was developed to measure energy expenditure by integrating accelerometry with multiple physiologic sensors including galvanic skin resistance, heat flux, body temperature and near body ambient temperature. The armband is worn 7 consecutive days on the right upper arm over the triceps muscle and monitors various physiological and movement parameters. The armband provides a reproducible and accurate measure in subjects with chronic illness with moderate functional limitations [57].

 8. Six questions are used to measure self-rated improvement after therapy and the satisfaction of the patient. The questions: ‘How satisfied are you with the effect of treatment’? ‘Is there a difference in how you handle problems now compared to before treatment started?’ and ‘Is there a difference in your daily activities now compared to your situation before treatment started?’ are scored on a 5-point Likert scale (‘1’ is very content/much improvement and ‘5’ is very discontented/situation is worse). The question: ‘To what extend did you achieve your personal treatment goals?’ is scored on a 10-point Likert scale, range 1–10. The questions: ‘Would you recommend the treatment to other CFS patients?’ can be answered with ‘yes’, ‘no’ or ‘I don’t know’. The question: ‘Do you still consider yourself a CFS patient?’ can be answered with ‘yes’ or ‘no’ [58].

 9. Life satisfaction will be measured by the Life Satisfaction Questionnaire, Dutch version (LSQ-DV). The LSQ-DV has one question on general life satisfaction and eight questions about domain-specific life satisfaction: self-care ability, leisure situation, vocational situation (including housekeeping), financial situation, sexual life, partner relationship, family life, contacts with friends and acquaintances. Questions are answered on a 6-point Likert scale (‘1’ is very dissatisfied, ‘6’ is very satisfied). The reliability of the LSQ-DV has been proven to be moderate to good for most domains in a patient group with chronic illness [59].

 10. Quality of life and utilities (health-related quality of life) will be measured by means of the standard Dutch version of the EuroQol (EQ-5D) [60]. The EQ-5D contains five dimensions of health-related quality of life, namely mobility, self-care, daily activities, pain/discomfort and depression/anxiety. Each dimension can be rated at three levels: no problem, some problems and major problems. The five dimensions can be summed into a health state. Utility values are calculated for these health states, using preferences elicited from a general population, the so-called Dolan algorithm [61]. The utility values derived from the Dolan algorithm will be used to compute Quality Adjusted Life Years (QALYs). The Dolan algorithm has been established using a general population from the UK. Also a Dutch algorithm has become available that will be used in the sensitivity analysis [62].

#### Treatment expectancy and credibility

Patients’ initial beliefs about the success of a given treatment have been shown to have an important influence on the final treatment outcome. A study by Smeets et al. (2008) [63] found evidence of predictive validity of expectancy and credibility scored by patients with chronic low back pain before following different active interventions To measure treatment expectancy and credibility, 2 weeks after the start of treatment, all participants will be asked to fill in the Dutch version of the Devilly and Borkovec questionnaire [64].

#### Mediation

In order to understand how treatment works, mediation analyses are performed. Two studies on mediation could not confirm the mediating role of physical activity in reducing fatigue in CFS. Moss-Morris (2005) [21] investigated physical activity as a mediator in the treatment effect of GET and Wiborg (2010) [65] in CBT. Since physical activity does not mediate the outcome, other parameters have to be responsible for a decrease of fatigue during therapy. In the model of Vercoulen et al. [14], somatic attributions, focussing on pain and fatigue, and low self-efficacy contribute to the perpetuating of CFS complaints. Patients with CFS feel helpless and surrendered to their complaints, making it difficult to change their situation. During therapy patients learn to change cognitions into more helpful cognitions, which increases self-efficacy. They learn to accept their situation in the present, making choices based on helpful cognitions and bodily symptoms other than pain and fatigue, and learn to focus on getting better instead of focussing on complaints and somatic attributions. Our hypothesis is that by increasing self-efficacy, decreasing somatic attributions and increasing the present-centred attention-awareness during activities first, behavioural changes can be made based upon these changes and eventually the severity of fatigue will decrease.

There are no studies that we are aware of that determine the mediating role of self-efficacy, somatic attributions and present-centred attention-awareness. Mediation will be investigated by the three-step method described by Baron and Kenny [66]. Before treatment (T1), 6 and 14 weeks after start of treatment (T2, T3), CIS, SE28, CAL and MAAS are filled in by the patient in order to analyse mediation at different moments during treatment phase.

#### Cost analysis

The Trimbos/iMTA questionnaire for Costs associated with Psychiatric Illness (Tic-P) will be used to measure treatment costs and additional expenses [67]. The subsection on absence from work (productivity loss by absenteeism and by loss of productivity while at work, and informal care and domestic help) is filled in every month. The subsection on health care costs (medical treatments, paramedic therapy, alternative therapy, self-care groups, clinical or outpatient treatment in hospital and other institutions, and medication) is filled in every 3 months. Treatment hours are registered by the therapists and the rehabilitation physicians in checklists filled in after each treatment session. The valuation of health-care costs, patient and family costs will be based on the updated Dutch manual for cost analysis in health-care research [68]. For care for which no costs-guidelines are available, estimations of the costs will be made based on the real costs or on population-based estimates from the literature.

### Assessment and procedures

After inclusion, the research assistant contacts the patient to make an appointment for the baseline assessment (T1). During T1 the patient is asked to wear the activity monitor for one week and to fill in the following questionnaires: 

 Checklist Individual Strength (CIS)

 Short Form 36 (SF-36)

 EuroQol- 5D (EQ-5D)

 Symptom Check List-90 (SCL-90)

 Self-Efficacy Scale-28 (SES 28)

 Causal Attribution List (CAL)

 Mindfulness Attention Awareness Scale (MAAS)

 Sickness Impact Profile-8 (SIP-8)

 Life Satisfaction Questionnaire, Dutch Version (LSQ-DV)

The patient is instructed on how to fill in the Tic-P, part II on health- and non-health-related costs every month for 1 year. One week after baseline assessment, the research assistant collects the activity monitor. The research assistant gives the patient a blind envelope with the treatment conditions. Treatments start within 4 weeks. Two weeks after the start of treatment, a patient is asked to fill in the list of Borkovec and Devilly and the PSCG, part 1. At 3 and 9 months after T1 a patient is asked to fill in the Tic-P, part I at home. Six months after the start of treatment the research assistant assesses the patient again (T4). The patient is asked to wear the activity monitor and fill in the same questionnaires as in T1 completed by the PSCG, part II self-rated improvement questionnaire and Tic-P, part I. Twelve months after the start of the treatment, the same assessment as in T4 will be repeated (T5). For each patient the study takes 12 months.

### Randomisation

After signing the informed consent form the patients are randomly divided into two groups: CBT and MRT. For each rehabilitation centre a randomisation list was generated by computer under supervision of an independent statistician. Before recruitment of patients an independent person prepared sealed envelopes for each rehabilitation centre and numbered them sequentially according to the randomisation list. The envelope is given to the participating patient by an independent research assistant after baseline assessment. The patient is asked not to open the envelope in front of the research assistant. Randomisation is performed for each centre to prevent differences between the treatment groups in the distribution of centres.

### Adverse events

Patients are able to contact a physician, who is appointed as an independent physician for the study, at any time. Adverse events will be monitored carefully. If any adverse event occurs or a patient withdraws from treatment, the researcher or research assistant will ask the patient and the therapist(s) why the patient is withdrawing. If deterioration is reported, the patient is offered appropriate help if needed. Referrals to other institutions are registered. Each patient withdrawing from treatment is asked to participate in the follow-up measurements. Patients who are not willing to participate in the follow-up measurements are asked to fill in questionnaires at home without wearing the activity monitor. Reasons for not wanting to participate in the follow-up measurement are registered.

### Analyses

#### Sample size

Based on the available literature [25,34,69] and our pilot study, mean CIS-fatigue scores at the start of CBT or multidisciplinary rehabilitation are about 50–52 with a standard deviation of 3.9-5.9. Following previous trials [28,34], we assume that a difference of 0.5 SD of the mean group score at baseline is clinically relevant. This equals a difference of about 3.0 points on the CIS fatigue scale. With a sample size of 48 patients in each treatment arm, accepting an alpha error of 0.05 and a power of 0.80, it is possible to measure a minimal difference of 3.0 points on the CIS fatigue scale. To compensate for an estimated 25% dropout rate, a total of 120 people will be included.

#### Analyses of efficacy

The effects of therapy conditions on the various outcomes will be compared using an ‘intention to treat’ approach. Data will be analysed with mixed linear regression models. The follow-up measurement will be the dependent variable, and the baseline value of the particular outcome will be added as covariate as well as random intercepts for individuals to allow for dependence within patients and centres [70]. Effect modification will be evaluated by introducing interactions between therapy condition and the potential modifiers in the equation. There will be a post-hoc analysis of the non-response group and the missing values. Dropout patients will be asked about the reason for stopping treatment or not attending a measurement. Patient characteristics of the dropouts will be compared to those of the group that completed each treatment. For the analyses we will use SPSS statistical software.

#### Economic analyses

Health-care costs will be measured using the Tic-P. Total costs are calculated by using an update of the Dutch manual for costing in economic evaluations [68]. Clinical outcomes 12 months after the start of treatment will be used in the economic evaluation. Student’s *t*-test for statistical significance will be used to measure differences between MRT and CBT. Fatigue severity during 1 year of follow-up will be used as the primary outcome measure for cost-effectiveness.

A cost-utility analysis will be performed by relating the mean total costs to the mean health utility (EQ-5D) scores of both groups. The costs per QALY of both treatments will be compared. Our primary (base-case analyses) will be performed according to the intention-to-treat principle, including data from all participants regardless of whether they received the intervention or not. For the analyses we will use SPSS statistical software and Excel (for the bootstraps).

Respondents for whom at least 75% of the data per measurement instrument are available will be included in the analysis. Missing data on item level will be handled using SPSS missing value analysis. Completely missing measurements will be handled using multiple imputation (MI). A baseline analysis will be performed to examine the comparability of groups at baseline for both costs and outcomes. If necessary, methods will be applied to control for differences in baseline [71]. To investigate whether data are normally distributed, a Kolmogorov-Smirnov test will be performed. Despite the usual skewness in the distribution of costs, the arithmetic means will be generally considered the most appropriate measures to describe cost data [72,73]. Non-parametric bootstrapping is a method based on random sampling with replacement based on individual data of the participants [74]. The bootstrap replications will be used to calculate 95% confidence intervals around the costs (95% CI), based on the 2.5th and 97.5th percentiles. If cost data are distributed normally, *t*-tests will be used.

The incremental cost-effectiveness ratio (ICER) will be determined on the basis of incremental costs and the effects of the MRT in comparison with CBT. The cost-effectiveness ratio will be expressed in terms of costs per unit of outcome; the cost-utility ratio will focus on the incremental cost per QUALY gained. The robustness of the ICER will be checked by non-parametric bootstrapping. Bootstrap simulations will also be conducted in order to quantify the uncertainty around the ICER, yielding information about the joint distribution of cost and effect differences. The bootstrapped cost-effectiveness ratios will be subsequently plotted in a cost-effectiveness plane in which the vertical line reflects the difference in costs and the horizontal line reflects the difference in effectiveness. The choice of treatment depends on the maximum amount of money the society is prepared to pay for a gain in effectiveness, which is called the ceiling ration. Therefore, the bootstrapped ICERs will also be depicted in a cost-effectiveness acceptability curve showing the probability that MRT is cost-effective using a range of ceiling ratios. Additionally, to demonstrate the robustness of our base-case findings, a multi-way sensitivity analysis will be performed.

#### Mediation analyses

In the mediation analysis, we investigate whether self-efficacy, somatic attributions and/or present-centred attention-awareness intervenes in the relationship between treatment and outcome. Multiple regressions are used to explore which factors mediate the outcome. Mediation is suggested when the change in the putative mediating factors is significantly related to treatment as the independent variable, outcome is significantly related to treatment as the independent variable, and finally, the relationship between outcome and treatment decreases (or goes to zero) when the change in the mediation factor is entered into the equation [66].

#### Participant non-adherence with treatment

Participant non-adherence with treatment will be measured both by recording attendance and by therapist ratings of adherence to therapy.

#### Trial management and oversight

The day-to-day management of the trial is carried out by the principal investigator, Desirée Vos-Vromans, in consultation with other members of the trial team and the research assistants. Every rehabilitation centre has one or two research assistants.

## Discussion

Treatment facilities for patients with CFS in multidisciplinary rehabilitation settings are rare, because most health insurance companies and rehabilitation centres are not convinced of the benefit of MRT for this group. MRT in this form is unique and has never been investigated in a multicentre RCT. The results of the FatiGo trial will provide information on the effects of CBT and MRT, mediators of the outcome, cost-effectiveness, cost-utility and the influence of treatment expectancy and credibility on the effectiveness of both treatments in patients with CFS.

### Trial status

At the time of the first submission of the manuscript, data collection was ongoing. Currently the data collection has been completed. The data will be analysed from May until October 2012. Results of the trial will be available in November 2012.

## Competing interests

The authors declare that they have no competing interests.

## Authors’ contributions

All authors contributed to the overall design of this study and are involved in the ongoing management of the trial. DV is the principal investigator with overall responsibility for the FatiGo trial. RG and LR participated in developing the multidisciplinary rehabilitation treatment, training and supervising the therapists and rehabilitation physicians. SE developed the health economic analysis plan. All authors read and approved the final article.
